# Effect of SMILE-derived decellularized lenticules as an adhesion barrier in a rabbit model of glaucoma filtration surgery

**DOI:** 10.1186/s12886-021-02090-3

**Published:** 2021-09-09

**Authors:** Houfa Yin, Xinyi Chen, Xiaogang Hong, Jian Ma, Fang Wu, Ting Wan, Yiwen Sang, Qiuli Fu, Zhenwei Qin, Danni Lyu, Wei Wu, Jinfu Yin, Yabo Yang

**Affiliations:** 1 Eye Center, the Second Affiliated Hospital, Zhejiang University School of Medicine, Hangzhou, China; 2 Department of Ophthalmology, People’s Hospital of Kaihua, Kaihua, China; 3 Department of Laboratory Medicine, the Second Affiliated Hospital, Zhejiang University School of Medicine, Hangzhou, China

**Keywords:** SMILE, Decellularized lenticule, Adhesion barrier, Glaucoma, Trabeculectomy

## Abstract

**Background:**

To investigate the effects of small incision lenticule extraction (SMILE)-derived decellularized lenticules on intraocular pressure (IOP) and conjunctival scarring in a rabbit model of glaucoma filtration surgery.

**Methods:**

Trabeculectomy was performed on both eyes of New Zealand rabbits. A decellularized lenticule was placed in the subconjunctival space in one eye of the rabbits (the decellularized lenticule group), and no adjunctive treatment was performed in the fellow eye (the control group). The filtering bleb features and IOP were evaluated 0, 3, 7, 14, 21, and 28 days after surgery, and histopathologic examination was performed 28 days after surgery.

**Results:**

Decellularized lenticules significantly increased bleb survival and decreased IOP postoperatively in the rabbit model with no adverse side effects. The histopathologic results showed a larger subconjunctival space and less subconjunctival fibrosis in the decellularized lenticule group.

**Conclusions:**

Decellularized lenticules can prevent postoperative conjunctiva-sclera adhesion and fibrosis, and they may represent a novel antifibrotic agent for trabeculectomy.

## Background

Glaucoma is one of the leading causes of irreversible blindness worldwide. Lowering of intraocular pressure (IOP) remains the only proven treatment to slow the progression of glaucoma [1]. Trabeculectomy, in which a drainage bypass is created to allow excess aqueous humour to drain into a conjunctival filtering bleb, is one of the most effective glaucoma filtration surgeries for reducing IOP [2]. However, filtration bleb dysfunction often occurs due to excessive scar tissue formation at the surgical site [3]. To reduce excessive scar formation, antimetabolic agents such as mitomycin C (MMC) and 5-fluorouracil (5-FU) are often used during trabeculectomy surgery and have been shown to improve the surgical outcome [4, 5]. However, these antimetabolic agents may lead to serious postoperative complications, such as persistent postoperative hypotony, corneal toxicity, filtering bleb leakage, blebitis, and endophthalmitis [4, 5]. Thus, a more physiological approach to suppressing subconjunctival fibrosis is needed.

Several investigations have been conducted to prevent bleb adhesion and fibrosis using physical barriers that are placed in the subconjunctival space or underneath the scleral flap. These include hyaluronate hydrogels, biodegradable polymers, and nonbiodegradable polymers [5–7]. Recently, small incision lenticule extraction (SMILE) has been proven to be a safe, efficient, and predictable corneal refractive surgery [8, 9]. With an increasing number of patients undergoing SMILE, the extracted lenticules could be used for various purposes in the ophthalmic field, such as hyperopia correction, keratoconus treatment, and the management of corneal perforation [10–12]. Decellularized lenticules are thin and transparent and exhibit good biocompatibility *in vivo *[13]. Therefore, we suggest that decellularized lenticules can act as a physical adhesion barrier during trabeculectomy surgery.

The aim of this study was to evaluate the efficacy of SMILE-derived decellularized lenticules for reducing adhesions between the conjunctiva and sclera, and keeping the filtering blebs active after trabeculectomy in rabbit eyes.

## Materials and methods

The use of SMILE-derived lenticules was approved by the Ethics Committee of the Second Affiliated Hospital, Zhejiang University School of Medicine, and the procedures used conformed to the tenets of the Declaration of Helsinki. Male New Zealand white rabbits (weighing 2-2.5 kg, aging 3–4 months) were supplied by the Academy of Medical Sciences of Zhejiang Province. All animal experiments were approved by the Animal Ethics Committee of the Second Affiliated Hospital, School of Medicine, Zhejiang University and were conducted in accordance with the Association for Research in Vision and Ophthalmology (ARVO) statement for the use of animals in ophthalmic and vision research. This study was performed in compliance with the ARRIVE guidelines. The IRB number is 2021 − 0551.

### Decellularization of the SMILE-derived lenticules

**Fig. 1:**
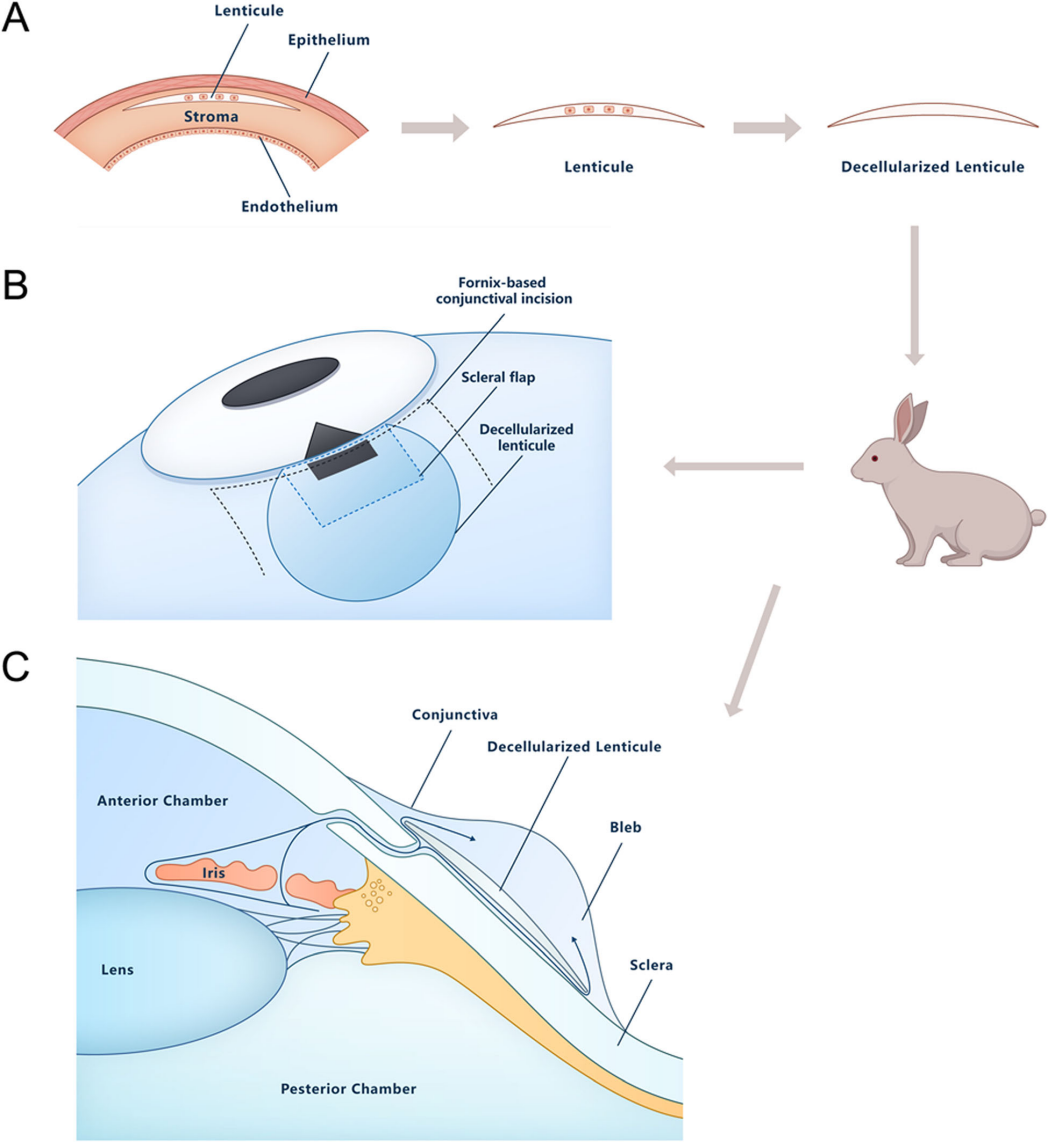
Schematic illustration of a decellularized lenticule for the prevention of subconjunctival fibrosis after trabeculectomy. **A** Decellularization of the SMILE-derived lenticule. **B** Schematic diagram illustrating the surgical procedures used to place the decellularized lenticule. **C** Schematic depiction of the concept to prevent subconjunctival fibrosis after trabeculectomy

SMILE-derived lenticules were collected during refractive surgery using the VisuMax femtosecond laser system (Carl Zeiss Meditec AG, Jena, Germany) as described in our previous study [13]. Lenticules with a diameter of 6.6 mm and a central thickness of < 50 μm were selected for the following procedures. The fresh lenticules were decellularized using sodium chloride (NaCl) and nucleases and cryopreserved at -80 °C in a balanced salt solution containing 50 mg/mL penicillin, 50 mg/mL streptomycin, 100 mg/mL neomycin, and 2.5 mg/mL amphotericin as described in our previous study (Fig. 1A) [13].

### Surgical procedure

As different rabbits had significantly different baseline IOP and wound-healing reactions, surgery was performed on both eyes of the rabbits. After creating the scleral flap, the eye was randomly assigned to the decellularized lenticule group or the control group. Twelve eyes of 6 rabbits were used in this study. The rabbits were anaesthetized with an auricular vein injection of sodium pentobarbital (30 mg/kg), and topical anaesthesia using 0.4 % oxybuprocaine hydrochloride eye drops was administered before surgery. Trabeculectomy was then performed with previously reported methods by an experienced glaucoma specialist (J.F.Y) with a few modifications [14]. Briefly, a fornix-based flap of conjunctiva was carefully dissected and a 3 × 3 mm partial thickness scleral flap was separated. After a 1 × 2 mm trabecular tissue was removed, peripheral iridectomy was performed. The scleral flap was not sutured, but the conjunctiva was closed with a 10 − 0 nylon suture in both groups. In the decellularized lenticule group, the decellularized lenticule was loosely secured by suturing on the sclera with 10 − 0 nylon (Fig. 1B). Only trabeculectomy was conducted on the control group, and no decellularized lenticule was placed.

### Clinical evaluation

After topical anaesthesia, the IOP was measured by Tono-pen (Reichert, Depew, NY, USA) at baseline and 3, 7, 14, 21, and 28 days after surgery.

The Tono-pen has been proven to provide accurate IOP measurements in rabbits after topical anaesthesia with excellent intrasession repeatability, excellent inter-operator reproducibility, and good intersession reproducibility [15–17]. It was equipped with an Ocu-Film tip cover and calibrated according to the manufacturer’s manual prior to the first use each day. Meanwhile, all IOP measurements were performed at the same time each day by the same investigator. An average of three measurements taken from each eye was recorded. The bleb appearance was examined via a slit lamp and was graded as previously described at 3, 7, 14, 21, and 28 days after surgery [18]. The blebs were graded from 0 to 4+, indicating increasing bleb height and size as follows: 0, no observable bleb; 1+, minimal height, conjunctiva thickening, and no microcysts; 2+, microcysts present but covering less than 75° of the eye; 3+, high bleb covering 75 to 135° of the eye; and 4+, greatly elevated bleb covering more than 135° of the eye [18].

### Histological analysis and immunohistochemistry

The rabbits were euthanized 28 days after surgery by an overdose intravenous injection of sodium pentobarbital. The eyeballs were enucleated and fixed in 4 % paraformaldehyde solution overnight. Then the eyeballs were dissected at the equator and embedded in paraffin. Four-micrometer-thick serial sections were cut through the centre of the operation site and stained with haematoxylin and eosin (H&E) for general histologic examination. Masson trichrome staining was performed to evaluate scar tissue formation. To examine the myofibroblasts adjacent to the surgical site, we immunohistochemically measured the expression of α-smooth muscle actin (α-SMA).

### Statistical analysis

Each measurement was expressed as the mean ± standard deviation (SD). The Mann-Whitney *U* test and an unpaired *t*-test were used to compare the bleb scores and IOP between the 2 groups. A *P* value less than 0.05 was considered statistically significant. All analyses were performed using Statistical Package for the Social Sciences software (version 22.0, International Business Machines Corp.)

## Results

### Slit-lamp examination and bleb appearance

**Fig. 2:**
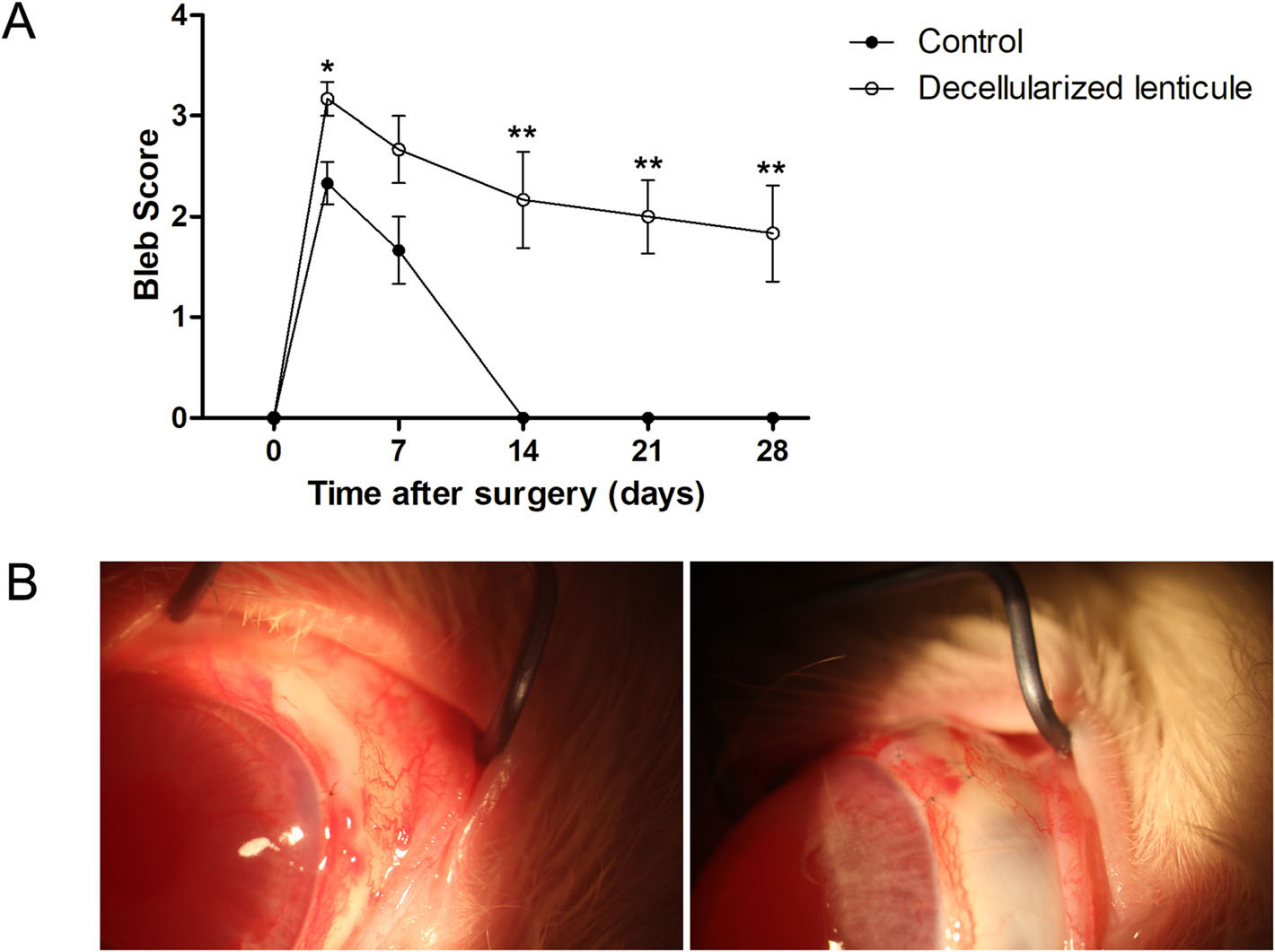
Bleb scoring via slit-lamp examination. **A** Bleb score changes in the control group and the decellularized lenticule group. *P < 0.05, **P < 0.01 versus the control group. **B** Representative photographs of blebs in the control group and the decellularized lenticule group 7 days after surgery

Slit-lamp examination revealed no severe postoperative inflammation in the anterior chamber, and no bleb leakage, blebitis, or endophthalmitis was observed during the postoperative period in either group. The bleb morphology was scored based on its appearance and size at 3, 7, 14, 21, and 28 days after surgery [18]. Filtering blebs were maintained over the scleral flap in the decellularized lenticule group for at least 28 days, whereas the filtering blebs collapsed within 14 days after surgery in the control group. The bleb scores were significantly higher in the decellularized lenticule group than in the control group on day 3 and from day 14 to day 28 (*P* < 0.05, Fig. 2).

### Postoperative IOP changes

**Fig. 3:**
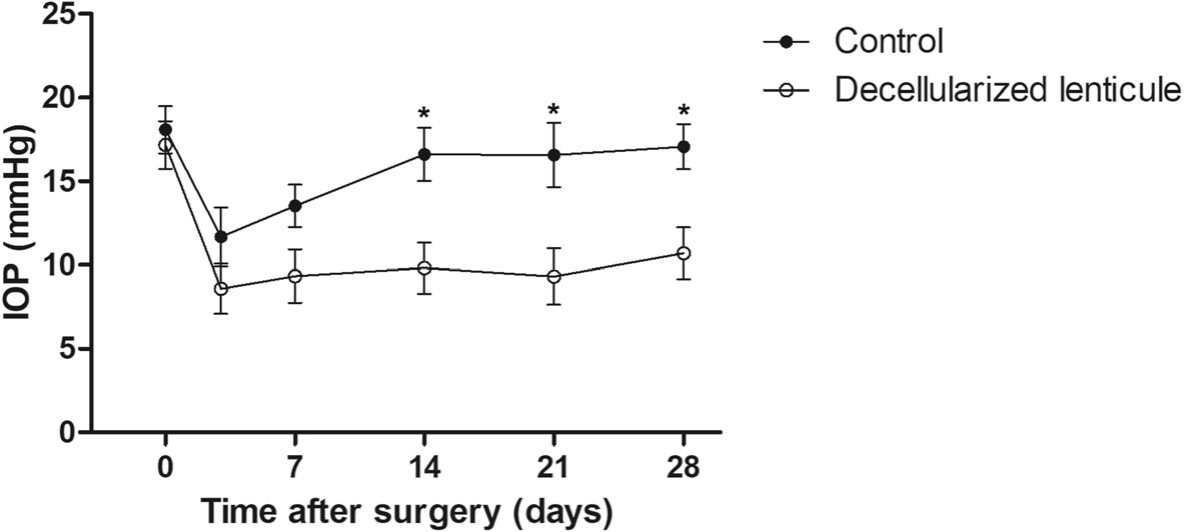
IOP changes in the control group and the decellularized lenticule group. *P < 0.05 versus the control group

There was no significant difference in the initial IOP between the decellularized lenticule group and the control group (Fig. 3). The IOP was reduced 3 days after surgery in both groups, and it did not differ significantly between the two groups within 7 days after surgery (Fig. 3). However, the IOP began to increase again in the control group 7 days after surgery, and the IOP of the decellularized lenticule group was significantly lower than that of the control group from day 14 to day 28 (*P* < 0.05, Fig. 3).

### Histopathologic features

**Fig. 4:**
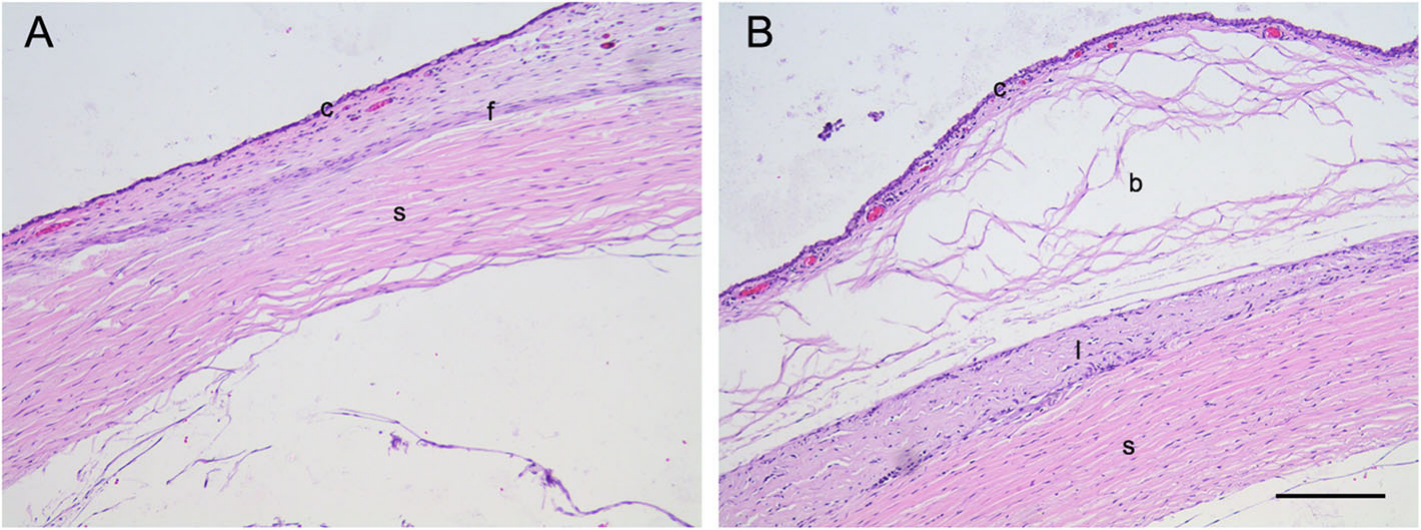
Histologic characteristics of the filtration site stained with H&E 28 days after the surgery. **A** The control group. **B** The decellularized lenticule group. Intact conjunctiva epithelium and prominent filtering space with small amounts of fibroblasts were observed in the decellularized lenticule group; however, the filtering space disappeared, and scar tissues were significantly deposited in the subconjunctival area in the control group. c, conjunctiva; b, subconjunctival space; f, scar tissues; l: decellularized lenticule; s, sclera. Scale bar: 100 μm

**Fig. 5:**
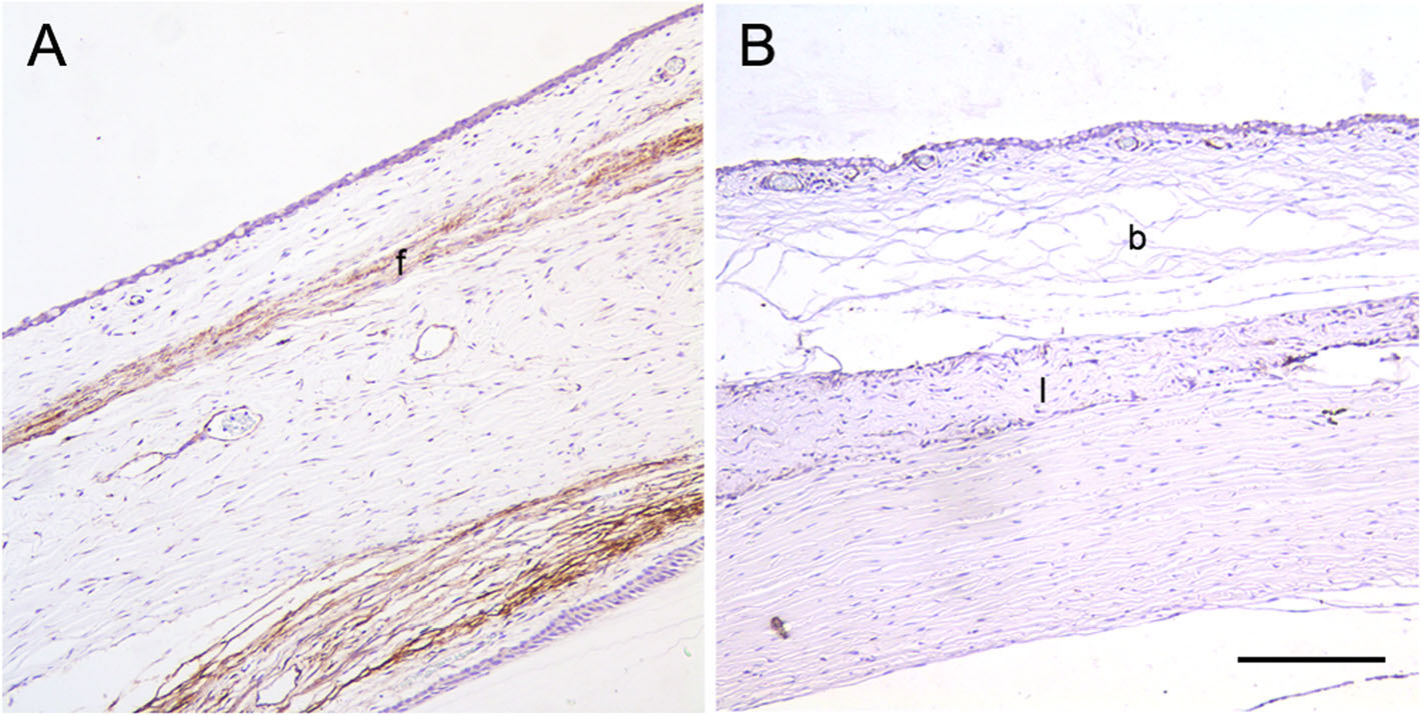
Immunohistochemical staining for α-SMA in the filtration site 28 days after surgery. **A** The control group. **B** The decellularized lenticule group. The subconjunctival area showed increased expression of α-SMA in the control group and the filtering spaces were not recognized. In contrast, only a few positive-staining cells were observed in the decellularized lenticule group, and filtering spaces were formed. b, subconjunctival space; f, α-SMA positive-staining cells; l: decellularized lenticule; s, sclera. Scale bar: 100 μm

**Fig. 6:**
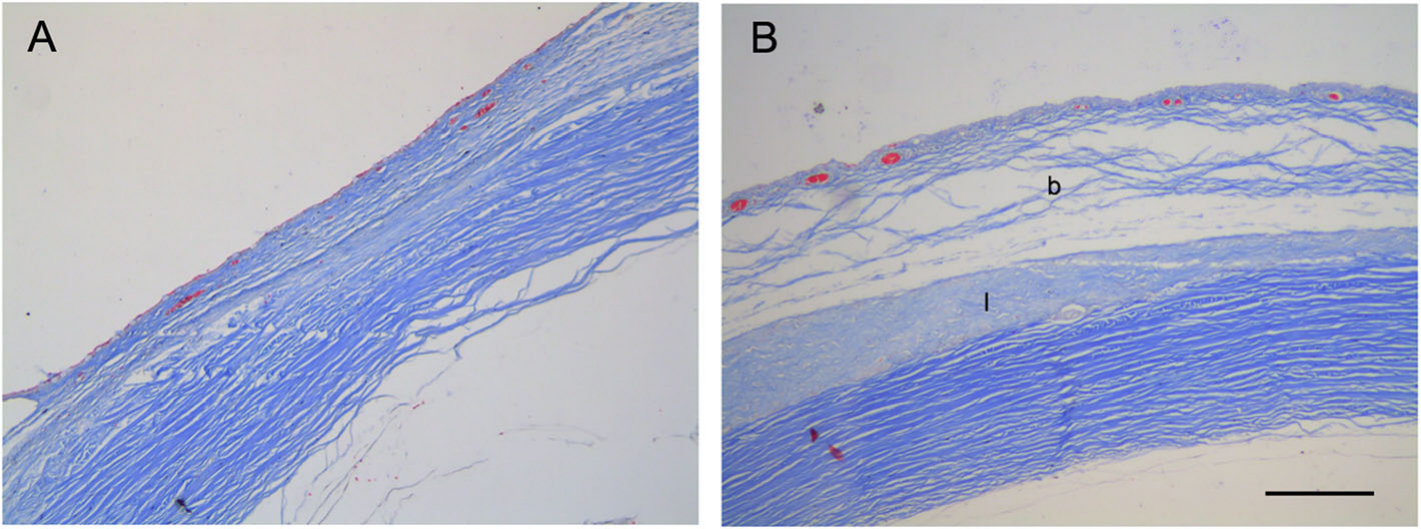
Histologic characteristics of the filtration site stained with Masson’s trichrome 28 days after surgery. **A** The control group. **B** The decellularized lenticule group. The subconjunctival area showed significant collagen deposition in the control group. However, less collagen deposition was observed in the decellularized lenticule group. b, subconjunctival space; l: decellularized lenticule. Scale bar: 100 μm

Histopathologic examination was performed 28 days after surgery to evaluate the effects of decellularized lenticules on bleb scarring. H&E staining revealed that the filtering space between the conjunctiva and lenticule remained prominent in the decellularized lenticule group while no filtering space was observed in the control group; however, massive scarring was observed in the control group (Fig. 4 A and B). No evidence of obvious inflammatory changes or tissue damage was observed in either group (Fig. 5B). To assess the degree of the subconjunctival fibrotic response, we performed immunohistochemical staining for α-SMA (a marker of myofibroblasts). Many cells with intensive α-SMA expression were observed in the subconjunctival area in the control group, indicating severe fibrosis (Fig. 5A). However, bleb fibrosis was significantly attenuated in the decellularized lenticule group (Fig. 5B). Consistent with α-SMA expression, Masson’s trichrome staining demonstrated significant collagen deposition in the subconjunctival region of the control group (Fig. 6A). In contrast, there was less collagen deposition in the decellularized lenticule group (Fig. 6B).

## Discussion

The present study demonstrates for the first time that the use of decellularized lenticules for trabeculectomy in rabbits keeps the filtering bleb active and maintains IOP reduction by inhibiting the formation of subconjunctival fibrosis.

Antimetabolites such as MMC and 5-FU are commonly used during trabeculectomy to inhibit subconjunctival fibrosis [4, 5]. However, the usage of these antimetabolites has been associated with higher risks for wound healing disorders and severe infections due to their non-selectivity [4, 5]. In recent years, adhesion barriers between the conjunctiva and sclera have been investigated as alternative methods for preventing bleb adhesion and fibrosis, including PDMAA polymer, expanded polytetrafluoroethylene (Gore-Tex) membrane, seprafilm, biodegradable collagen, and honeycomb-patterned film [5–7, 14]. Although some of these adhesion barriers have proven effective in reducing bleb adhesion and fibrosis in animal models, clinical trials have shown inconsistent results concerning surgical outcomes [5].

SMILE has become clinically available as an alternative to laser in situ keratomileusis since 2011 [19, 20]. The extracted lenticule is the immediate by-product of this procedure and it is typically discarded after the surgery. The increasing popularity of this surgery has made it easier to obtain SMILE-derived lenticules. The decellularized lenticule is a thin stromal layer with low immunogenicity and good biocompatibility, making it an excellent candidate for corneal stromal regeneration [21, 22]. We have previously shown that decellularized lenticules could safely and effectively repair damage to the anterior cornea in rabbits [13]. Recently, Gu et al. also reported that subretinally transplanted decellularized lenticules exhibited excellent biocompatibility without obvious adverse reactions and fibrosis [23]. Therefore, decellularized lenticules might be a useful biomaterial in various types of ophthalmic surgery.

The present study revealed that decellularized lenticules promote IOP reduction and prolong bleb survival in trabeculectomy in rabbits with no complications. A strategy for reducing scar formation following glaucoma filtration surgery is to reduce the adhesion of tenon fibroblasts to the underlying sclera at the surgical site [24]. Our *in vivo* studies suggest that decellularized lenticules have a space-keeping effect that prevents adhesion between tenon fibroblasts and sclera (Fig. 1C). To fix the decellularized lenticule precisely in the desired area, it was loosely sutured onto the sclera. However, we speculate that there is a passage between the sclera and the decellularized lenticule that diverts aqueous humour from the anterior chamber to the subconjunctival space, given that IOP reduction and bleb formation were observed in the postoperative period (Fig. 1C).

Myofibroblast accumulation and excessive collagen deposition in the subconjunctiva are major causes of bleb failure [25]. The histopathologic examination showed that myofibroblasts infiltrated the subconjunctival area with compact collagen deposition in the control group; however, fewer myofibroblasts and less collagen deposition were observed in the subconjunctiva in the decellularized lenticule. Limitations of our study include the small sample size and the short follow-up duration, which may limit the power of this study. Additional studies with larger sample sizes and longer follow-ups are warranted to clarify the safety and efficacy of decellularized lenticules in glaucoma filtering surgery.

## Conclusions

In conclusion, decellularized lenticules may effectively inhibit excessive scar formation in glaucoma filtering surgery. Our study provides a novel separating agent to prevent subconjunctival fibrosis after trabeculectomy and to increase the success rate of glaucoma filtering surgery.

## Data Availability

The datasets used and/or analyzed during the current study are available from the corresponding author on reasonable request.

## Abbreviations

5-FU: 5-fluorouracil
α-SMA: α-smooth muscle actin
ARVO: Association for Research in Vision and Ophthalmology
H&E: Hematoxylin and eosin
IOP: Intraocular pressure
MMC: Mitomycin C
NaCl: Sodium chloride
SD: Standard deviation
SMILE: Small incision lenticule extraction
